# 573. CD388, a Novel Drug-Fc Conjugate (DFC), Demonstrates Prophylactic Activity in an Influenza Human Challenge Model

**DOI:** 10.1093/ofid/ofae631.174

**Published:** 2025-01-29

**Authors:** Ozlem Equils, Shawn Flanagan, Sy-Shi Wang, Johan Vingerhoets, Wilbert van Duijnhoven, Alex James Mann, Roxana E Rojas, Taylor Sandison

**Affiliations:** Cidara Therapeutics, San Diego, California; Cidara Therapeutics, San Diego, California; Janssen Pharmaceutical, Brisbane, California; Janssen Pharmaceutica NV, Infectious Diseases and Vaccines, Beerse, Brussels Hoofdstedelijk Gewest, Belgium; Janssen Pharmaceutica NV, Beerse, Brussels Hoofdstedelijk Gewest, Belgium; hVIVO, London, England, United Kingdom; Janssen Research & Development, Brisbane, California; Cidara Therapeutics, San Diego, California

## Abstract

**Background:**

Influenza is a significant cause of morbidity and mortality globally. CD388 is a multivalent conjugate of a dimeric zanamivir stably linked to a proprietary human IgG1 Fc fragment engineered for extended half-life. In preclinical and two Phase 1 studies CD388 has been shown to be safe. Here we describe a post-hoc analysis of prophylactic activity of CD388 against influenza disease in a Phase 2a human challenge study.

**Figure:**
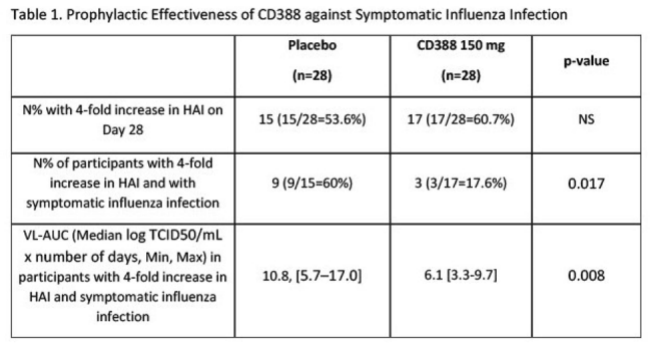

**Methods:**

Healthy participants (18-55 yrs) were randomized to receive subcutaneous (SQ) CD388 or placebo. CD388 was administered 5 days before intranasal challenge with A/Perth/16/2009 (H3N2). A hemagglutinin inhibition assay (HAI) was performed at the onset of quarantine, on Day 0 (prior to inoculation) and on Day 28. A 4-fold increase in Day 28 HAI titer with respect to Day 0 titer was considered as confirmation of influenza infection. Participants completed a graded symptom scoring system 3 x daily. Symptomatic infection was defined as RT-PCR-confirmed (2 quantifiable measurements on ≥ 2 independent samples over 2 days) or culture confirmed (1 quantifiable TCID50 measurement) influenza infection from Day 1 (pm) to Day 8 (am) AND fever or ≥ 2 symptoms at a single time point or any symptom of grade ≥ 2 at a single time point. Prophylactic efficacy was assessed among participants who experienced a 4-fold increase in HAI titer.

**Results:**

28 participants received 150 mg of CD388 and 28 received placebo. 17 participants in the CD388 arm (60.7%) and 15 participants in the placebo arm (53.6%) had a 4-fold increase in the HAI titer on Day 28 (Table 1). Although these end-points were not powered, of those with the 4-fold change in HAI, the number of symptomatic participants was significantly lower in the CD388 arm (3/17, 17.6%) compared to placebo (9/15, 60%; p= 0.017), and among these symptomatic participants the median AUC TCID50 (log TCID50/mL x number of days) was significantly lower in the CD388 arm (6.1, min 3.3, max 9.7) compared to placebo (10.8, min 5.7, max 17; p=0.008, Table 1).

**Conclusion:**

A single SQ dose of CD388 administered 5 days prior to influenza challenge was effective in preventing symptomatic disease. A Phase 2b clinical influenza prevention study for CD388 will begin in the Fall of 2024.

**Disclosures:**

**Ozlem Equils, MD**, Cidara Therapeutics: Employee|Cidara Therapeutics: Stocks/Bonds (Public Company) **Shawn Flanagan, PhD**, Cidara Therapeutics: Salaried Employee|Cidara Therapeutics: Employee|Cidara Therapeutics: Stocks/Bonds (Public Company)|Cidara Therapeutics: Stocks/Bonds (Public Company) **Sy-Shi Wang, Ph.D.**, Janssen Research & Development LLC: Employee|Janssen Research & Development LLC: Stocks/Bonds (Public Company) **Johan Vingerhoets, PhD**, Janssen Pharmaceutica NV: Stocks/Bonds (Public Company) **Wilbert van Duijnhoven, n/a**, Janssen Pharmaceutica NV: Employee|Janssen Pharmaceutica NV: Stocks/Bonds (Public Company) **Alex James Mann, MSc**, hVIVO: Employee **Roxana E. Rojas, M.D., Ph.D.**, Janssen Research & Development LLC: Employee|Janssen Research & Development LLC: Stocks/Bonds (Public Company)|Johnson and Johnson: Stocks/Bonds (Public Company) **Taylor Sandison, MD, MPH**, Cidara Therapeutics Inc: Employee|Cidara Therapeutics Inc: Stocks/Bonds (Public Company)

